# Efficacy and hypnotic effects of melatonin in shift-work nurses: double-blind, placebo-controlled crossover trial

**DOI:** 10.1186/1740-3391-6-10

**Published:** 2008-10-29

**Authors:** Khosro Sadeghniiat-Haghighi, Omid Aminian, Gholamreza Pouryaghoub, Zohreh Yazdi

**Affiliations:** 1 Baharloo Hospital, Tehran University of Medical Sciences, Tehran, Iran

## Abstract

**Background:**

Night work is associated with disturbed sleep and wakefulness, particularly in relation to the night shift. Circadian rhythm sleep disorders are characterized by complaints of insomnia and excessive daytime sleepiness that are primarily due to alterations in the internal circadian timing system or a misalignment between the timing of sleep and the 24-h social and physical environment.

**Methods:**

We evaluated the effect of oral intake of 5 mg melatonin taken 30 minutes before night time sleep on insomnia parameters as well as subjective sleep onset latency, number of awakenings, and duration of sleep. A double-blind, randomized, placebo-controlled crossover study with periods of 1 night and washouts of 4 days comparing melatonin with placebo tablets was conducted. We tried to improve night-time sleep during recovery from night work. Participants were 86 shift-worker nurses aged 24 to 46 years. Each participant completed a questionnaire immediately after awakening.

**Results:**

Sleep onset latency was significantly reduced while subjects were taking melatonin as compared with both placebo and baseline. There was no evidence that melatonin altered total sleep time (as compared with baseline total sleep time). No adverse effects of melatonin were noted during the treatment period.

**Conclusion:**

Melatonin may be an effective treatment for shift workers with difficulty falling asleep.

## Background

There is substantial evidence that the prevalence of sleep disorders is an important occupational health problem, especially among health care professionals on night or on rotating work shifts [1-10]. An important aspect of the work environment of nurses is that they are required to work at any point in the 24 hour day [11]. Night work is associated with disturbed sleep and impaired alertness. The impact of sleep is the result of the circadian interference with sleep during daylight hours and circadian suppression of pineal gland by light at night [12].

The definition of insomnia is a complaint of disturbed sleep, manifested as difficulties in sleep initiation, sleep maintenance, early morning awakenings, or nonrestorative sleep. Many sources also add the presence of associated daytime impairments, such as fatigue, irritability, decreased memory and concentration, and pervasive malaise affecting many aspects of daytime functioning [13]. In a recent study, 32% of night-shift workers reported symptoms of insomnia or excessive daytime sleepiness, whereas these symptoms were reported in only 18% of day workers from the same sample population [14]. Several studies have reported that sleep problems are more common among health care personnel on rotating work shifts [15]. The quality of sleep has been found to be altered in hospital nurses working on rotating shift schedules, especially those working morning and night shifts as well as in hospital nurses working shifts when compared with day nurses [16].

The pineal hormone melatonin regulates a variety of physiological processes, including circadian, cardiovascular, reproductive, and neuroendocrine functions [17-19]. However, it is the hypnotic effects of melatonin that are considered an integral component of its physiological role in sleep modulation [20,21]. Administration of melatonin facilitates sleep onset and improves the quality of sleep [22-24]. Administration of melatonin can also produce phase shifts in circadian rhythms, and has been used to treat the symptoms of circadian maladaptation associated with shift work [25-27]. There is currently a great deal of interest in whether properly timed melatonin administration can facilitate circadian phase shifting in these situations [28].

Normally, production of melatonin by the pineal gland is stimulated by darkness and inhibited by light [29]. Hajak et al [30] reported lower levels in blood melatonin in patients with insomnia than in healthy controls, and Haimov et al [31] found lower levels of urinary 6-sulphatoxymelatonin in elderly patients with insomnia than in healthy elderly subjects. Surprisingly, only a few studies have explored the beneficial effect of melatonin administration on night shift workers in an actual workplace setting [26]. The purpose of this study was to compare the efficacy of melatonin (5 mg) and placebo in the treatment of shift workers with insomnia.

## Methods

### Study Design

We conducted a double-blind, placebo-controlled, randomized crossover trial among healthy, non-smoking, non-pregnant shift worker nurses at Imam Hospital (Tehran, Iran). Each subject signed an informed consent document after the procedures had been fully explained. All subjects were asked to sign the consent form, confirming that they understood the goals, risks, and potential benefits of the study and their right to withdraw from the study at any time. The Ethical Committee of Tehran University approved the study.

Consenting participants were randomized to one of two sequences: placebo followed by melatonin or melatonin followed by placebo. The randomization list was completed using a random number generator. The treatment phase of each sequence consisted of taking a 5 mg tablet of melatonin about 30 minutes before habitual nighttime sleep. The placebo phase consisted of taking an identical looking placebo on the same schedule. Both melatonin and placebo were taken on the first night after night work. All participants had 3 visits to the hospital. At the first visit, eligibility was checked, participants gave informed consent, and baseline insomnia parameters were assessed by using seven questions [32]. Participants were asked to scale their difficulties in falling asleep, staying asleep and waking up too early with scores from 1 (no problem) to 5 (very severe problem). They were also asked to specify their sleep quality (satisfaction with their sleep) using scores from 1 (very satisfied) to 5 (very unsatisfied) and were asked to answer the following three questions about their habitual night time sleep: 1) How long does it take you to fall asleep? 2) How many times do you wake up during the night? 3) How many hours do you sleep?

All the participants who reported sleep problems in the baseline questionnaire were included in the study. In the first visit, one placebo or one melatonin was given to the nurses. They were asked to use it at home on the first night after shift work, about 30 minutes before their intended sleep. On the following morning, upon awakening, each participant answered the questionnaire. In the second visit, after the 4 days of washout and receiving their completed questionnaire, patients entered into another study period, conducted as in the first. In the third visit, the last completed questionnaire was received from nurses.

### Data Analysis

We used SPSS 11.5 for Windows for statistical analysis. When the Kolmogorov-Smirnov test confirmed normality, parametric tests were conducted. One-way analysis of variance was used to determine the statistical significance of subjective scores of insomnia from each phase of the trial. Statistically significant results detected by analysis of variance (*p *< 0.05) were further analyzed by using Tukey post hoc paired comparisons. Results in the text are expressed as mean +/- standard deviation.

## Results

Eigthy-six out of 118 participants completed the study. Eleven subjects dropped out during the first treatment phase because they could not attend the scheduled tablet taking, whereas one did not complete the second treatment phase because of an acute illness unrelated to the study protocol. Twenty participants did not report sleeping problems in the baseline questionnaire. Table 1 presents a description of the 86 subjects who completed the study. Females accounted for 80.2% of the participants, and the mean age was 30.05 years (range: 24–46 years). Subjective parameters of sleep obtained from the baseline questionnaire are shown in Table 1.

**Table 1:** Demographic and subjective sleep data of subjects (N = 86)

	Mean (SD) or percentage (n)
Sex	80.2% female (69)
Age	30.05 (5.2)
Mean BMI in kg/m2	26.7 (3.1)
Subjective sleep onset latency in min	37.5 (41.3)
Subjective number of awakenings	5.2 (2.1)
Subjective duration of sleep in min	450.5 (82.3)

Differences in sleep data at baseline, while taking placebo, and while taking melatonin are shown in Tables 2 and 3. Subjective sleep onset latency (SOL) was 37.5 +/- 41.3 minutes at the baseline. There was evidence of an effect of melatonin treatment on SOL. Specifically, the mean SOL for subjects being treated with melatonin was significantly lower than the mean SOL for subjects given placebo. Furthermore, means for both the subjects given melatonin and those given placebo were significantly different from the baseline mean (see Table 2). Although melatonin treatment did not significantly alter other insomnia variable compared with baseline values, there was a significant improvement in sleep quality with melatonin treatment (see Table 3).

**Table 2:** Subjective sleep parameters during a randomized, double-blind, placebo-controlled crossover study of shift work nurses: results at baseline and after 1 night melatonin or placebo treatment (*N *= 86)

		Mean	SD	P(vs.baseline^*a*^)	P(vs.placebo^*a*^)	F^b^	P^b^
Sleep onset latency	Melatonin	21.5	17.7	< 0.05	< 0.05	6.3	0.01
	Placebo	49.7	30.3	< 0.05			
	Baseline	37.5	41.3				
							
Total sleep time	Melatonin	392.1	52.4	> 0.05	> 0.05	0.49	NS
	Placebo	372	49.4	> 0.05			
	Baseline	450.5	82.3				
							
Number of awakenings	Melatonin	5.1	1.9	> 0.05	> 0.05	0.64	NS
	Placebo	5.1	1.9	> 0.05			
	Baseline	5.2	2.1				

a *p *values for Tukey post hoc analysis.

b Overall test for differences

**Table 3:** Subjective assessment of insomnia: results at baseline and after 1 night of melatonin or placebo treatment (*N *= 86)

		Mean	SD	P(vs.baseline^*a*^)	P(vs.placebo^*a*^)	F^b^	P^b^
Difficulty falling asleep	Melatonin	1.63	0.61	< 0.05	< 0.05	4.5	0.01
	Placebo	2.53	0.62	> 0.05			
	Baseline	2.67	0.80				
							
Difficulty staying asleep	Melatonin	2.32	0.83	> 0.05	> 0.05	0.71	NS
	Placebo	2.31	0.69	> 0.05			
	Baseline	2.48	1.11				
							
Problem waking up too early	Melatonin	2.26	0.81	> 0.05	> 0.05	0.42	NS
	Placebo	2.40	0.74	> 0.05			
	Baseline	2.39	1.29				
							
Sleep quality	Melatonin	2.58	0.76	< 0.05	< 0.05	1.2	0.02
	Placebo	2.69	0.67	> 0.05			
	Baseline	3.16	0.92				

a *p *values for Tukey post hoc analysis.

b Overall test for differences

## Discussion

In 86 shift-work nurses with insomnia disorders, administration of 5 mg of melatonin about 30 minutes before a night time sleep significantly decreased sleep onset latency (SOL) and increased sleep quality as compared with placebo. We observed a significant improvement in falling asleep induced by 5 mg of melatonin (16 min) as compared to baseline, which supports the well known capacity of this hormone to change biological rhythms [33-36].

Placebo was not equal to baseline for SOL. A general meta-analysis of placebo effects pointed to a nonsignificant beneficial effect on sleep latency (a 10-min decrease in subjective estimates of sleep latency) in five clinical trials [37]. Melatonin did not alter the other sleep parameters that we measured. These findings are in accordance with those of earlier open trials using smaller numbers of subjects [38,39].

The focus of our analysis was a comparison of assessment of insomnia parameters (sleep onset, sleep maintenance, sleep quality) in nurses with shift working at baseline, after a one-night melatonin treatment, and after a one-night placebo treatment. Our study revealed no major impact of melatonin on difficulty staying asleep or waking up too early. Our results agree with a previous investigation suggesting that patients with primary insomnia have a pathophysiologic disturbance that is not reversed by melatonin [40]. No adverse effects of melatonin were noted during the treatment.

The fact that we observed a reduction in SOL but no change in sleep duration after melatonin administration has at least two possible explanations: either 1) melatonin caused a small (16 min) phase advance of the circadian system or 2) random variation obscured a correspondingly small lengthening of total sleep time. Our data do not provide the basis for favoring either one or the other of these alternatives.

A limitation of this study is that we were unable to perform polysomnography or actigraphy to evaluate sleep parameters objectively. Our results based on subjective self-reports were, however, very encouraging. Regarding the high prevalence of insomnia in shift workers, more studies about melatonin effect on different kinds of insomnia parameters (difficulty falling asleep, difficulty staying asleep, problem waking up too early, and sleep quality) causing by shift working is recommended.

## Conclusion

Melatonin may be an effective treatment for shift workers with difficulty falling asleep.

## Competing interests

The authors declare that they have no competing interests.

## Authors' contributions

KHS helped with the conception and design of the study, helped to supervise the staff and helped to draft the manuscript. OA and GHP helped with the conception and design of the study and helped draft the manuscript. ZY helped with the conception and design of the study, helped to collect the data, participated in the data analyses and interpretation, and helped draft the manuscript. All authors read and approved the final manuscript.
